# Creating smoke free environments by local policy

**DOI:** 10.1093/eurpub/ckac129.035

**Published:** 2022-10-25

**Authors:** S Rouwenhorst, A Koornstra

**Affiliations:** GGD GHOR Nederland, Utrecht, Netherlands

## Abstract

In The Netherlands, around 20.000 people die each year from the effects of smoking and passive smoking. Every week, hundreds of children become addicted to smoking. To improve the overall public health in the Netherlands, it is important to substantially reduce the prevalence of smokers. Until recent years, municipalities played next to no role in helping to reduce smoking in the Netherlands. The start of a large campaign that aims a smokefree generation by 2040 has changed that. Part of that campaign is to create smoke-free environments. In a two-year program, 90% of the Dutch municipalities started policy and actions to ban smoking from public places, as part of the National Prevention Agreement. The participating municipalities were supported by all 25 Regional Health Services (GGDs) by providing knowledge and manpower on a regional level, together with national, regional and local organisations. The project has a number of successful elements: the GGD received a (very small) budget to actively participate in the region; knowledge was shared between the regions and from national to local level and legal issues were dealt with by the Nat Assoc of Municipalities (VNG). In 2021, 317 out of 352 Dutch municipalities were active in this project. There is a significant increase of smoke-free environments, mostly schoolyards, playgrounds, sports facilities, smoke-free bus stops and municipal institutions. Other results are integrated plans for smoking cessation care and accessible stop smoking service, e.g. in GGD Fryslan the number of registrations for smoking cessation care increased dramatically and 80% actually quit smoking. All parties involved agree that the strength of this project is its positive approach and the broad social support it generates for the Smokefree Generation. It is about persuasion, not compulsion. Working from a national focus towards a smoke-free environment, while taking into account local differences and needs, makes this project a success.

**Key messages:**

• This project resulted in more local and regional partnerships and an increase of smoke-free environments, bringing the ultimate goal of a Smokefree Generation closer.

• 90% of Dutch municipalities have created smoke-free environments with the guidance of the regional GGD, financed by Ministry of Health: multilevel governance works.

